# Case Report of a Rapidly Progressive Indolent T‐Cell Lymphoma of the Gastrointestinal Tract

**DOI:** 10.1002/deo2.70340

**Published:** 2026-05-19

**Authors:** Keitaro Shibuya, Akiko Tamura, Daiki Yafuso, Ami Kawamoto, Shuji Hibiya, Kento Takenaka, Hiromichi Shimizu, Toshimitsu Fujii, Kazuo Ohtsuka, Ryuichi Okamoto

**Affiliations:** ^1^ Department of Gastroenterology and Hepatology Institute of Science Tokyo Tokyo Japan; ^2^ Department of Human Pathology Institute of Science Tokyo Tokyo Japan; ^3^ Endoscopic Unit Institute of Science Tokyo Hospital Tokyo Japan

**Keywords:** balloon‐assisted enteroscopy, chronic diarrhea, indolent T cell lymphoma of the gastrointestinal tract, small intestine, ulcer

## Abstract

Indolent T‐cell lymphoma of the gastrointestinal tract (iTCL‐GI) is a relatively newly recognized disease entity. iTCL‐GI was first proposed by Perry et al. in 2013, before it was formally incorporated as a distinct disease category in the fifth edition of the World Health Organization Classification of Hematolymphoid Tumors: Lymphoid Neoplasms in 2022. Although the prognosis of iTCL‐GI is generally favorable, some cases may present with malignant transformation or severe clinical manifestations. Herein, we describe a case of iTCL‐GI with a poor clinical outcome. An 85‐year‐old female presented with chronic diarrhea and weight loss. Esophagogastroduodenoscopy and colonoscopy performed at the referring clinic were nondiagnostic, and the patient was referred to our institution. Balloon‐assisted enteroscopy revealed multiple ulcerative lesions in the distal ileum, which were eventually diagnosed as iTCL‐GI based on the biopsy findings. Palliative care was provided in accordance with the patient's preferences. Her gastrointestinal symptoms progressed rapidly, and she died 5 months after symptom onset. In this report, we discuss how balloon‐assisted enteroscopy can be useful in the diagnosis of iTCL‐GI, as well as a review of the literature on potential risk factors for poor clinical outcomes, including the presence of ulcers on endoscopy.

## 1 Introduction

Indolent T‐cell lymphoma of the gastrointestinal tract (iTCL‐GI) is a primary gastrointestinal monoclonal T‐cell lymphoproliferative disorder comprised of small, mature lymphocytes. This disease entity was first proposed by Perry et al. in 2013, based on a case series of 10 patients [1]. In 2022, iTCL‐GI was officially included as a distinct disease category in the fifth edition of the World Health Organization Classification of Hematolymphoid Tumors: Lymphoid Neoplasms [2]. Primary gastrointestinal lymphomas account for approximately 30%–40% of extranodal lymphomas; mostly of B‐cell origin. Meanwhile, T‐cell lymphomas are uncommon, accounting for <9% of cases [3]. Among the gastrointestinal T‐cell lymphomas, iTCL‐GI is particularly rare, with <80 cases reported to date [4], while its clinical behavior and optimal treatment strategy remain poorly established.

The symptoms of iTCL‐GI, which include abdominal pain, diarrhea, and weight loss, are nonspecific. Due to overlapping pathological features, the disease is often misdiagnosed as inflammatory bowel disease or celiac disease. In addition, patients commonly exhibit an indolent and stable clinical course over several years to decades [1]. Herein, we report a case of iTCL‐GI exhibiting a rapidly progressive clinical course, and a review of the relevant literature was performed.

## 2 Case Report

An 85‐year‐old Japanese female patient with a medical history of bronchiectasis, pulmonary tuberculosis, and angina presented with diarrhea occurring approximately 10 times per day. Esophagogastroduodenoscopy (EGD) and colonoscopy (CS) performed at her local clinic did not reveal any remarkable findings, and the patient was followed up with symptomatic treatment.

However, her diarrhea did not improve over the subsequent 3 months, and the patient experienced a weight loss of 10 kg. Thus, she was referred to our institution for further evaluation. She was then admitted as an inpatient for further assessment and treatment due to worsening nausea and decreased appetite.

**FIGURE 1:**
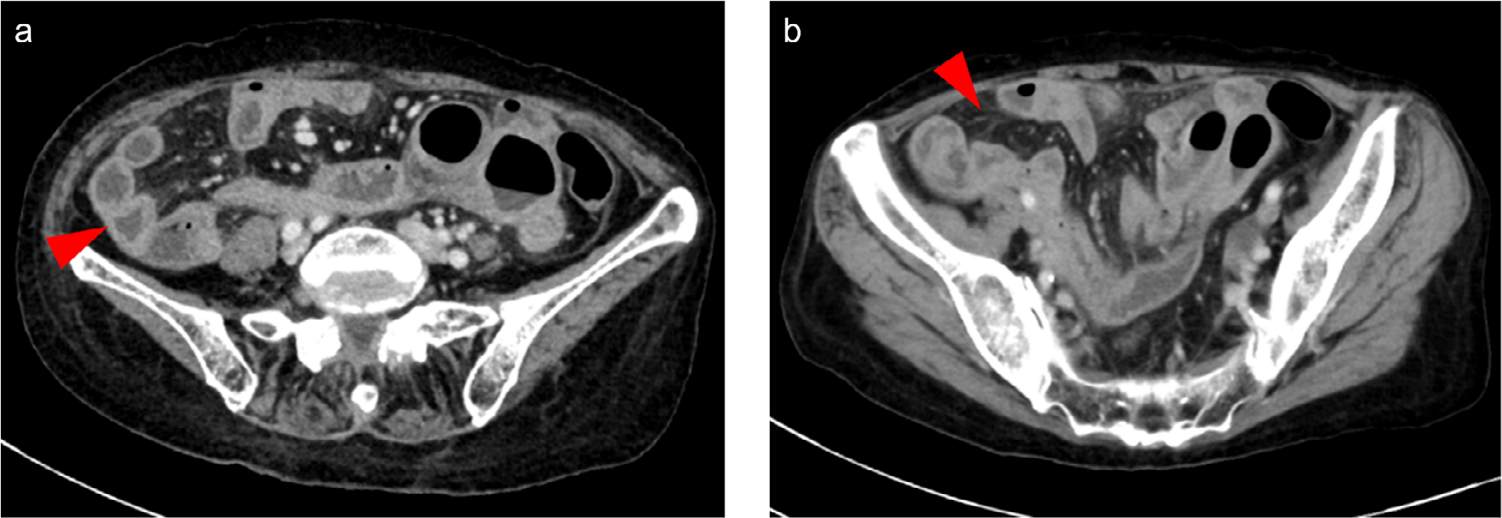
Abdominal computed tomography (CT) revealed (a) wall thickening of the small intestine with luminal fluid retention (red arrow) and (b) increased attenuation of the mesenteric fat (red arrow).

**FIGURE 2:**
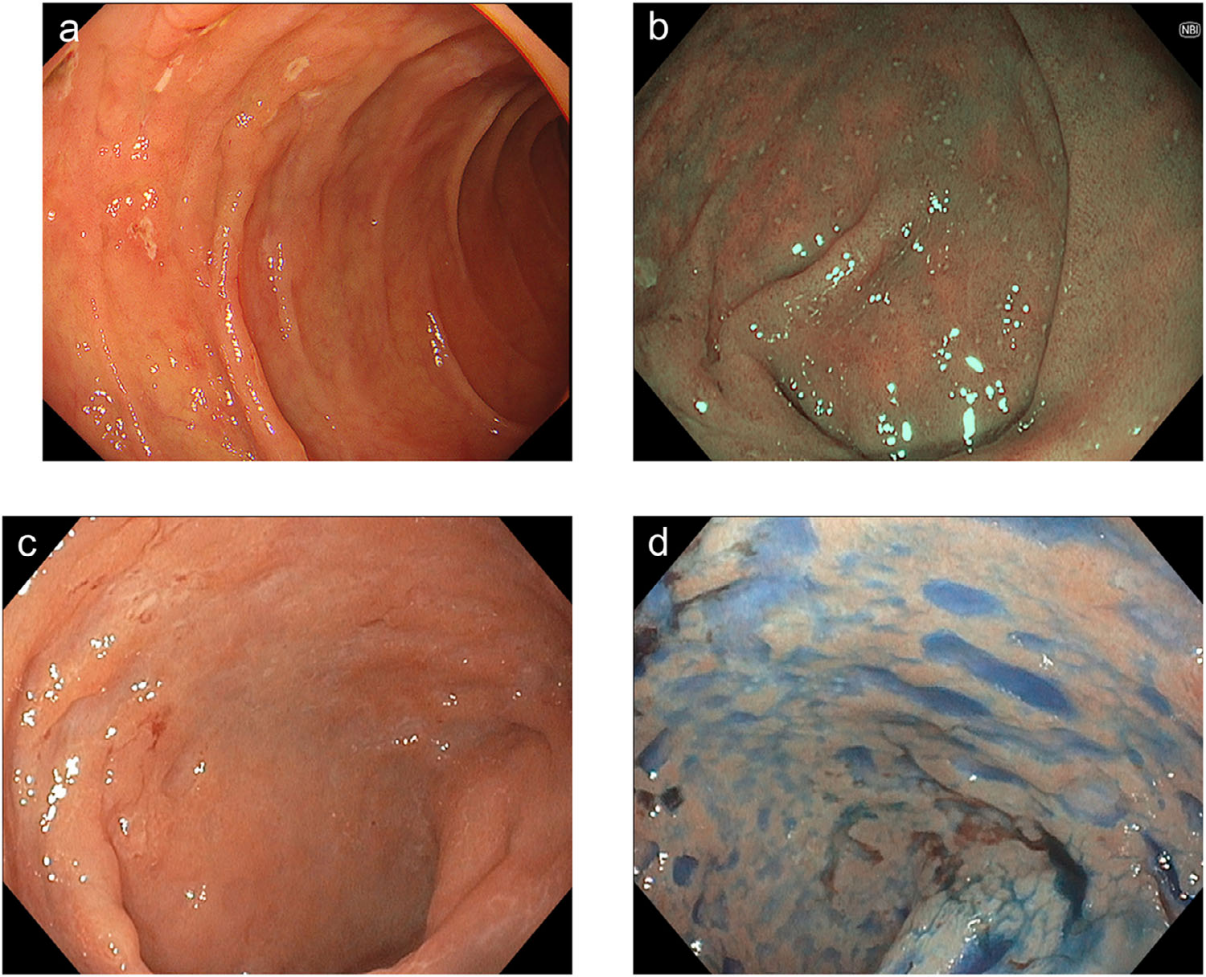
Endoscopic images of (a) duodenum, (b) sigmoid colon, and (c, d) distal ileum. Images (a) and (c) were obtained using white‐light imaging, image (b) using narrow‐band imaging, and image (d) after indigo carmine spraying. Esophagogastroduodenoscopy (EGD) revealed multiple shallow ulcers in the duodenum. Balloon‐assisted enteroscopy (BAE) demonstrated villous atrophy and multiple ulcerative lesions in the distal ileum. Aphthous lesions were also observed in the sigmoid colon.

**FIGURE 3:**
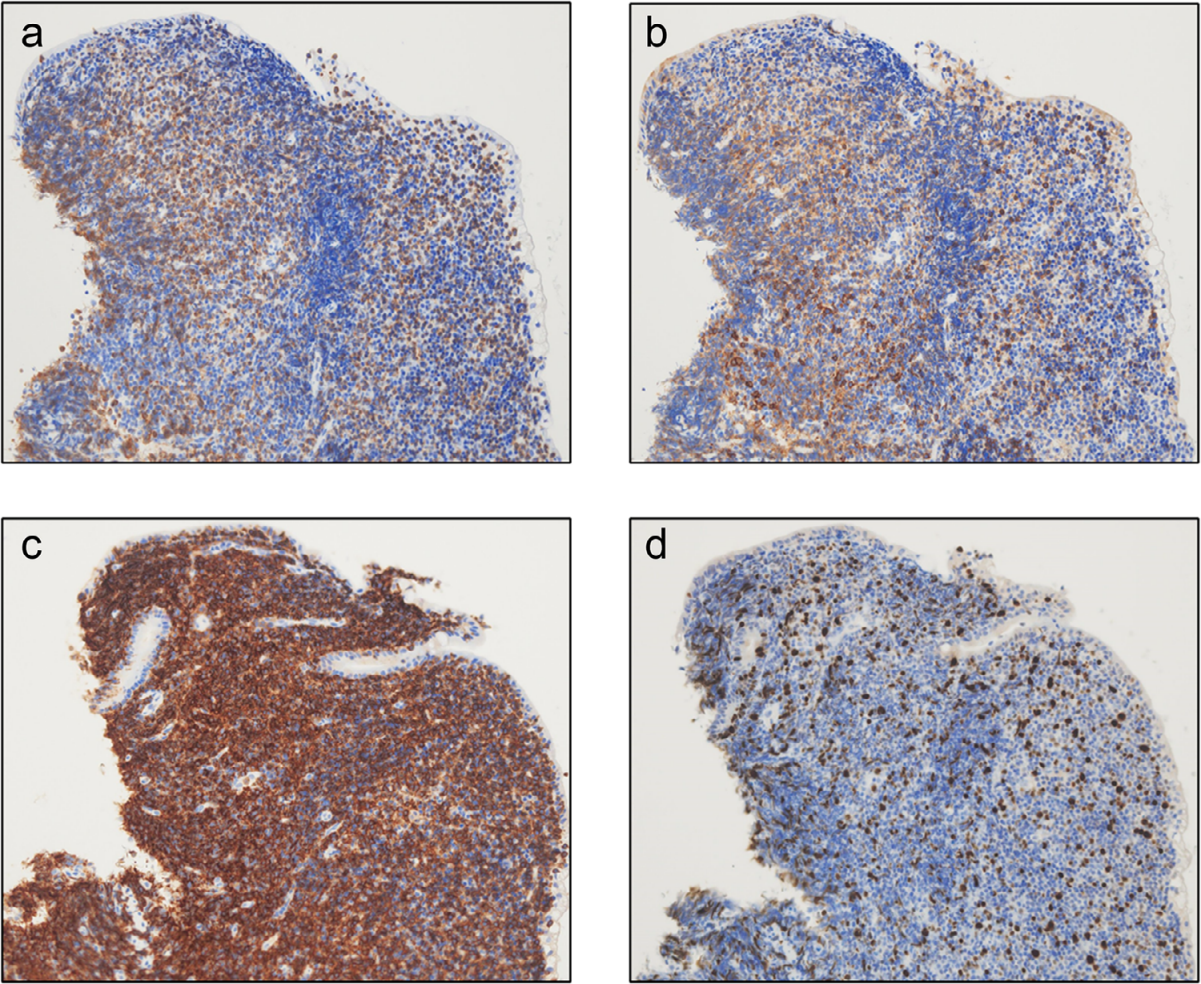
Immunohistochemical findings of the ileal biopsy at an original magnification of ×200. Weak positivity for (a) CD3 and (b) CD5 is observed. (c) Positivity for CD7 is observed. (d) The Ki‐67 labeling index is approximately 10%.

On physical examination, the patient was afebrile and had mild diffuse abdominal tenderness. Laboratory tests showed an elevated C‐reactive protein level (6.64 mg/dL), hypoalbuminemia with a serum albumin level of 2.7 g/dL, and mild anemia with a hemoglobin level of 9.7 g/dL. The soluble interleukin‐2 receptor level was significantly high at 4172.4 U/mL. The stool culture results were negative. Computed tomography (CT) scan revealed wall thickening of the small intestine with luminal fluid retention and increased attenuation of the mesenteric fat (Figure 1a,b). EGD revealed multiple shallow ulcers in the esophagus and duodenum (Figure 2a). In light of the CT scan findings, retrograde balloon‐assisted enteroscopy (BAE) was performed to further evaluate the small intestine. BAE showed villous atrophy and multiple ulcerative lesions extending from the terminal ileum to the distal ileum. Aphthous lesions were also observed in the sigmoid colon and rectum (Figure 2b–d). Histopathological examination of biopsy specimens collected from the distal ileum revealed loss of the villous architecture and infiltration of small lymphoid cells in the lamina propria. Immunohistochemical analysis showed weak positivity for CD3 and CD5, positivity for CD7, and a Ki‐67 labeling index of approximately 10% (Figure 3a–d). The patient tested weakly positive for CD4 and negative for CD8. The CD56 expression was low, CD20 was negative, and Epstein–Barr virus‐encoded RNA in situ hybridization was negative. Similar but less prominent pathological findings were also observed in the biopsy specimens obtained from the duodenum. Based on the endoscopic and histopathology findings, infectious enteritis, systemic inflammatory diseases such as collagen vascular disease, and inflammatory bowel disease were considered unlikely. Drug‐induced gastrointestinal injury was considered as a differential diagnosis. Thus, enteric‐coated aspirin, which the patient had been taking for chest discomfort, was discontinued. However, her symptoms did not improve.

According to the clinical course and pathological findings, the patient was diagnosed with iTCL‐Gi on hospital day 21. Treatment with corticosteroids was considered. However, the patient and her family declined aggressive therapy due to old age, and a palliative care approach was adopted. Her symptoms, including nausea and abdominal distension, progressively worsened. She gradually developed a persistent fever, and abdominal radiography revealed significant bowel dilatation with fluid retention. Eventually, the patient could not tolerate oral intake and was transferred to a palliative care unit on hospital day 33. After transfer, her symptoms, such as fever, nausea, abdominal distension, and diarrhea, persisted, and her serum albumin levels decreased to 1.5 g/dL. The patient experienced progressive general debilitation and died on hospital day 42.

## 3 Discussion

Generally, iTCL‐GI has a favorable prognosis [1]. A standard treatment strategy has not been established, and chemotherapeutic response is often limited [4]. Thus, cautious observation is frequently selected as the initial management approach. Nonetheless, in approximately 10% of reported cases, an aggressive clinical behavior, including transformation to high‐grade lymphoma and the development of severe complications, is observed [5]. In such cases, active therapeutic intervention may be required, thereby emphasizing the importance of identifying factors associated with poor prognosis. Several reports have shown that CD4‐positive cases may have a higher risk of disease progression than CD8‐positive cases [5]. However, the definitive risk factors for disease aggravation have not yet been completely elucidated.

In this case, the patient exhibited a rapidly progressive and fatal clinical course, and she died within 5 months of symptom onset. To the best of our knowledge, there are no previously reported cases of iTCL‐GI resulting in death within 6 months of disease onset. In our previously reported cases, as in those presented by Nagaishi et al., the patient achieved clinical remission and had a favorable prognosis [6]. In terms of endoscopic findings, the current case was characterized by multiple ulcerative lesions. Meanwhile, no ulcers were observed in the previously reported case, which only showed villous atrophy. High‐grade gastrointestinal lymphomas are often accompanied by deep ulcerative lesions, suggesting that ulcer formation may reflect a higher biological aggressiveness even in iTCL‐GI. [7] Therefore, we focused on endoscopic findings as potential indicators of poor prognosis and performed a review of the literature.

A search of PubMed using the terms “indolent T‐cell lymphoproliferative disorder of the gastrointestinal tract” or “indolent T‐cell lymphoma of the gastrointestinal tract” yielded 51 reported cases with available endoscopic findings. Among these, ulcerative lesions were observed in 16 cases. Of these, eight had poor outcomes, including cases requiring surgery due to severe complications such as perforation and bowel obstruction; cases showing transformation to or coexistence with high‐grade lymphoma; cases resulting in death; and cases with involvement of other organs (Table 1). In contrast, among the 35 patients without ulcerative lesions, only three experienced adverse events, including transformation to high‐grade lymphoma and bowel obstruction requiring surgical intervention. Out of the reported endoscopic findings in iTCL‐GI, including congestion, erythema, erosions, ulcers, and nodular lesions, the presence of ulcerative lesions may be considered a potential indicator of poor prognosis.

**TABLE 1:** Previous case reports of iTCL‐GI with poor prognosis.

First author (year)	Age (sex)	Endoscopic findings	Lesion	Symptoms	Treatment	Prognosis
A. M. Perry (2013) [1]	37 (M)	Ulceration and inflammation	Colon	Diarrhea, abdominal pain, and ulcers in the oral cavity	Chemotherapy	Developed aggressive T‐cell lymphoma and died of progressive disease
L. Guo (2019)	46 (M)	Diffuse small nodular hyperplasia, irregular ulcers, and intestinal stricture	Jejunum	Intermittent paraumbilical colic pain, bloating, and occasional diarrhea	Observation	Underwent surgery for small intestinal perforation and was diagnosed accompanied by diffuse large B‐cell lymphoma
J. Wu (2020)	42 (M)	A rough hyperemic mucosa and multifocal deep ulcers	Colon	Dental ulcers, abdominal pain, and diarrhea	Mesalamine	Accompanied by neck lymph node infiltration and new onset of classic Hodgkin's lymphoma
C. R. Soderquist (2020)	62 (M)	Mucosal nodularity, scalloping, mosaic pattern, increased vascularity, ulcer	Duodenum and jejunum	Diarrhea and weight loss	Chemotherapy	Died of small bowel perforation
R. David (2022)	52 (F)	Inflammatory polyp with ulceration, scalloping, and blunting of the villi	Stomach, duodenum, and terminal ileum	Abdominal pain, diarrhea, and weight loss	Budesonide	Developed dyspnea and chronic cough, and diagnosed pulmonary involvement of iTCL‐GI
C. Weng (2022)	45 (M)	Prominent congestion and edema, and multiple ulcers	Distal ileum and colon	Chronic diarrhea	Prednisone and azathioprine	Underwent surgery for small intestinal perforation
W. Fan (2023) [5]	52 (M)	Ulcers and thickened mucosa	Colon	Diarrhea, abdominal pain, and ulcers in the oral cavity	Chemotherapy	Died of disease progression
W. Fan (2023) [5]	58 (M)	Ulcers and thickened mucosa	Colon	Diarrhea, abdominal pain, and ulcers in the oral cavity	Chemotherapy	Died of disease transformation

Previous case reports of iTCL‐GI with poor prognosis.

Furthermore, among 51 cases, three were diagnosed using BAE, including the case reported by Guo et al., listed in the table, and the case reported by Nagaishi et al. [6]. In all three cases, the diagnosis was established by examining the small bowel using BAE after neither EGD nor CS identified the disease. It is speculated that in cases with mild symptoms, only EGD and CS are typically performed, and the disease may be overlooked. These findings emphasize the potential value of BAE in evaluating patients with unexplained chronic diarrhea.

In conclusion, we report a case of iTCL‐GI with an unexpectedly rapid and fatal clinical course. To date, the clinical features of iTCL‐GI have not been completely elucidated, and reports of rapidly progressive cases are limited. Nevertheless, such cases should be considered in future clinical practice. This case highlights the diagnostic role of BAE in unexplained chronic diarrhea and indicates a potential association between ulcerative endoscopic findings and poor prognosis.

## Author Contributions

*Conceptualization*: Keitaro Shibuya, Akiko Tamura, and Kazuo Ohtsuka. *Data Curation*: Keitaro Shibuya and Akiko Tamura. *Investigation*: Keitaro Shibuya and Akiko Tamura. *Project administration*: Keitaro Shibuya and Akiko Tamura. *Resources*: Keitaro Shibuya and Akiko Tamura. *Supervision*: Kazuo Ohtsuka and Ryuichi Okamoto. *Validation*: Keitaro Shibuya, Akiko Tamura, and Kazuo Ohtsuka. *Visualization*: Keitaro Shibuya and Akiko Tamura. *Writing – original draft preparation*: Keitaro Shibuya and Akiko Tamura. *Writing—revise & editing*: all authors. Approval of final manuscript: all authors.

## Funding

The authors have nothing to report.

## Ethics Statement

N/A.

## Conflicts of Interest

The authors declare no conflicts of interest.

## Data Availability

The data underlying this article are available in the article.
